# Circulating tRNA Fragments as a Novel Biomarker Class to Distinguish Acute Stroke Subtypes

**DOI:** 10.3390/ijms22010135

**Published:** 2020-12-24

**Authors:** T. Truc My Nguyen, M. Leontien van der Bent, Marieke J. H. Wermer, Ido R. van den Wijngaard, Erik W. van Zwet, Bas de Groot, Paul H. A. Quax, Nyika D. Kruyt, Anne Yaël Nossent

**Affiliations:** 1 Department of Neurology, Leiden University Medical Center, 2300 RC Leiden, The Netherlands; T.T.M.Nguyen@lumc.nl (T.T.M.N.); M.J.H.Wermer@lumc.nl (M.J.H.W.); I.R.van_den_Wijngaard@lumc.nl (I.R.v.d.W.); 2 Department of Vascular Surgery, Leiden University Medical Center, 2300 RC Leiden, The Netherlands; M.L.van_der_Bent@lumc.nl (M.L.v.d.B.); P.H.A.Quax@lumc.nl (P.H.A.Q.); 3 Einthoven Laboratory for Experimental Vascular Medicine, Leiden University Medical Center, 2300 RC Leiden, The Netherlands; 4 University Neurovascular Center Leiden-The Hague, 2300 RC Leiden, The Netherlands; 5 Neurology, Haaglanden Medical Center, 2501 CK The Hague, The Netherlands; 6 Department of Medical Statistics, Leiden University Medical Center, 2300 RC Leiden, The Netherlands; E.W.van_Zwet@lumc.nl; 7 Department of Emergency Medicine, Leiden University Medical Center, 2300 RC Leiden, The Netherlands; B.de_Groot@lumc.nl; 8 Laboratory Medicine, Medical University of Vienna, 1090 Vienna, Austria; 9 Internal Medicine II, Medical University of Vienna, 1090 Vienna, Austria

**Keywords:** acute stroke, biomarkers, small non-coding RNA, stroke codes, tRNA fragment, diagnostic accuracy

## Abstract

Early blood biomarkers to diagnose acute stroke could drastically reduce treatment delays. We investigated whether circulating small non-coding RNAs can serve as biomarkers to distinguish between acute ischemic stroke (IS), intracerebral hemorrhage (ICH) and stroke mimics (SM). In an ongoing observational cohort study, we performed small RNA-sequencing in plasma obtained from a discovery cohort of 26 patients (9 IS, 8 ICH and 9 SM) presented to the emergency department within 6 h of symptom onset. We validated our results in an independent dataset of 20 IS patients and 20 healthy controls. ICH plasma had the highest abundance of ribosomal and tRNA-derived fragments, while microRNAs were most abundant in plasma of IS patients. Combinations of four to five tRNAs yielded diagnostic accuracies (areas under the receiver operating characteristics curve) up to 0.986 (ICH vs. IS and SM) in the discovery cohort. Validation of the IS and SM models in the independent dataset yielded diagnostic accuracies of 0.870 and 0.885 to distinguish IS from healthy controls. Thus, we identified tRNA-derived fragments as a promising novel class of biomarkers to distinguish between acute IS, ICH and SM, as well as healthy controls.

## 1. Introduction

The diagnosis of acute stroke currently relies on clinical assessment and neuroimaging. While neuroimaging can rule out intracerebral hemorrhage (ICH), acute ischemic stroke (IS) can often not be confirmed and, therefore, diagnostic uncertainty can persist. Moreover, about 40% of suspected stroke patients presented to the emergency department as possible reperfusion candidates (so-called ‘stroke codes’) appear to have symptoms based on a different pathology than stroke: Stroke mimics (SM) [1]. It is critical to establish the diagnosis as quickly as possible, as the different types of stroke require completely different interventions. Particularly in acute IS speed is of the essence, as efficacy of reperfusion therapies sharply declines with time [2]. An early biomarker that reliably distinguishes stroke subtypes (IS or ICH) and SMs would therefore be of substantial clinical value.

Circulating small non-coding RNAs (sncRNAs) potentially fit this profile and especially microRNAs have been studied intensively in this context [3]. Recently, sncRNAs derived from transfer RNAs (tRNAs) have been described as an emerging biomarker class [4]. tRNAs are encoded in the human genome codes by more than 400 genes, which can be divided into 48 *isodecoders*: tRNAs with the same anticodon. Although tRNAs are best known for their role in protein synthesis, tRNAs and tRNA-derived fragments (tRFs) also play a role in regulatory processes such as gene expression and translational control [5]. Importantly, tRFs are found in liquid biopsies such as plasma and serum and show promise as biomarkers for several other pathologies, such as cancer and epilepsy [6,7].

We investigated whether sncRNAs in plasma, especially tRFs, can be used as early biomarkers to differentiate between acute IS, ICH and SM. 

## 2. Results 

### 2.1. Patient Characteristics

Our discovery cohort included 9 IS, 8 ICH and 9 SM patients, 58% of whom were men. The mean age was 71 ± 14 years, median onset-to-door-time was 78 min [IQR 57-116] and the median National Institutes of Health Stroke Scale (NIHSS) score of stroke patients was 8 [IQR 4-18] (Table 1). Patients with intracerebral hemorrhage more often had diabetes (*n* = 4; 50%) and hypertension (*n* = 7; 89%) compared to the ischemic stroke and stroke mimic group (*p* < 0.05).

### 2.2. Small RNAs in Plasma

**Figure 1:**
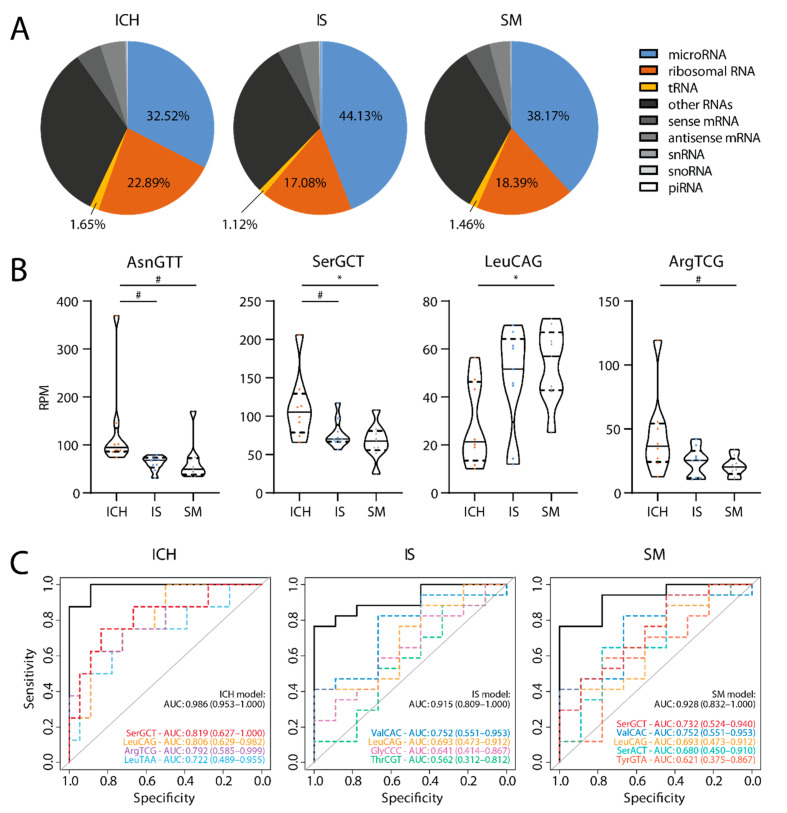
sRNA-seq results in the discovery cohort. (**A**) Distribution of reads assigned to various small non-coding RNAs (sncRNA) classes. (**B**) Summed tRNA-derived fragments (tRF) abundance of differentially expressed isodecoders. Tukey’s post-hoc test: # *p* < 0.10; * *p* < 0.05. (**C**) ROC analysis of diagnostic accuracy for each of the three groups from the other two groups. Dashed colored curves: Separate isodecoders in each optimal least absolute shrinkage and selection operator (LASSO) model. Black curves: Combination of these isodecoders. *n* = 8–9 per group. RPM: Reads per million; AUC: Area under the curve.

We detected RNAs from various sncRNA classes, including microRNA, ribosomal RNA and tRNA (Figure 1A). The distribution of the fraction of sequencing reads from these sncRNA classes differed between the groups. Notably, plasma of ICH patients contained the highest relative abundance of reads deriving from ribosomal RNAs and tRNAs (ICH: 22.89% and 1.65%; IS: 17.08% and 1.12%; SM: 18.39% and 1.46%, respectively), while microRNAs were relatively most abundant in IS plasma (IS: 44.13%; ICH: 32.52%; SM: 38.17%). tRFs showed the largest differences in abundance between the groups (ICH vs. IS: 1.47-fold; ICH vs. SM: 1.13-fold; SM vs. IS: 1.30-fold).

### 2.3. tRFs as Biomarkers

In addition to overall differences in tRF abundance, we found that several isodecoders were differentially expressed between the groups (*p* < 0.05; Figure 1B and Supplementary Table S1). LASSO analysis yielded models consisting of four to five isodecoders. By combining these different isodecoders in a single model, diagnostic accuracy was markedly increased over each of the individual isodecoders, reaching an area under the ROC curve (AUC) of 0.986 (95% CI, 0.953–1.000) for ICH, 0.915 (95% CI, 0.809–1.000) for IS and 0.928 (95% CI, 0.832–1.000) for SM vs. the other two groups (Figure 1C).

### 2.4. Validation of tRF Models

**Figure 2:**
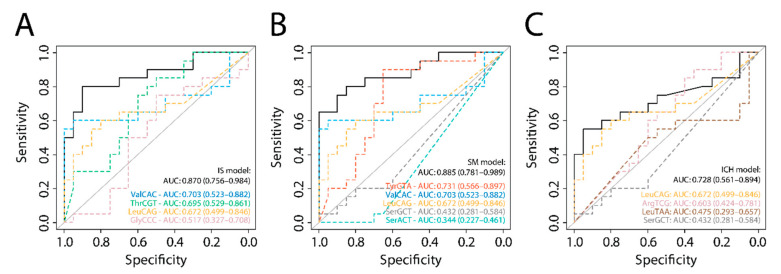
ROC analyses on the validation cohort. (**A**) Ischemic stroke (IS) model, (**B**) stroke mimic (SM) model and (**C**) intracerebral hemorrhage (ICH) model, as determined in our discovery cohort, applied to the sRNA-seq dataset from the validation cohort (IS vs. healthy controls; *n* = 20 per group). Dashed colored lines show the diagnostic potential of the separate isodecoders; black curves show the diagnostic potential of the combination of these. AUC: Area under the curve. HC: Healthy controls.

We validated these models using data from an independent cohort of IS patients and healthy controls (hereafter referred to as the validation cohort) [8]. Our models to distinguish IS and SM yielded comparable AUCs of 0.870 (95% CI 0.756–0.984) and 0.885 (95% CI 0.781–0.989) (Figure 2A,B and Supplementary Table S2). The validation cohort did not include ICH patients and accordingly, our ICH model had much lower diagnostic accuracy in this dataset (AUC: 0.728; 95% CI 0.561–0.894; Figure 2C). 

### 2.5. Common tRF Model

**Figure 3:**
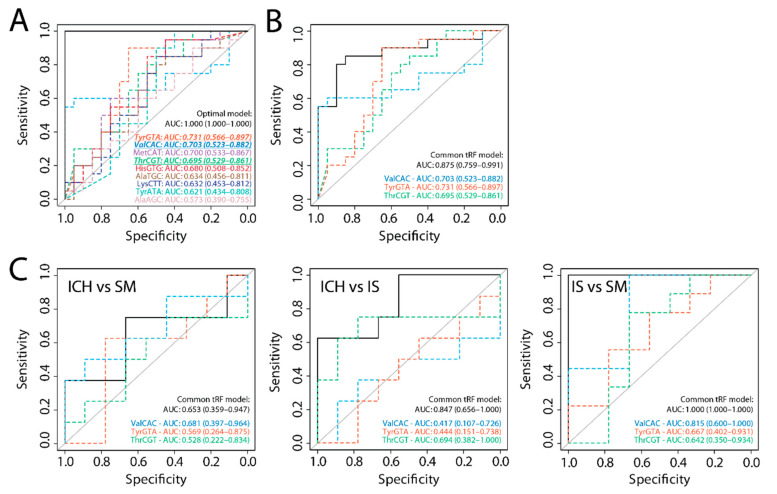
Common tRF model. (**A**) Optimal LASSO model determined in the validation cohort; the three tRF isodecoders that were identified in both the discovery and the validation cohort are italicized and underlined. (**B**) ROC analysis of these three common tRFs in the validation cohort. (**C**) Common tRF model applied to the discovery cohort (*n* = 8–9 per group) to distinguish between ICH and SM (left), ICH and IS (middle), and IS and SM (right). Dashed colored lines show the diagnostic potential of the separate isodecoders; black curves show the diagnostic potential of the combination of these. AUC: Area under the curve. HC: Healthy controls.

When an optimal LASSO model to distinguish IS from healthy controls was determined in the validation cohort, three out of nine isodecoders overlapped with our IS and SM models: tRNA-TyrGTA, tRNA-ThrCGT and tRNA-ValCAC (Figure 3A). The combination of these three isodecoders yielded an AUC of 0.875 (95% CI, 0.759–0.991) to distinguish IS from healthy controls in the validation cohort (Figure 3B and Supplementary Table S2). This common tRF model showed poor diagnostic accuracy in our discovery cohort to distinguish between ICH and SM (AUC 0.653; 95% CI, 0.359–0.947), but high diagnostic accuracy for distinguishing between IS and ICH (AUC 0.847; 95% CI, 0.656–1.000) and between IS and SM (AUC 1.000) (Figure 3C and Supplementary Table S2).

## 3. Discussion

We show that tRFs hold potential as biomarkers for the early diagnosis of acute stroke. Importantly, we found that tRF abundance from specific isodecoders can be used to (1) differentiate acute ICH from IS and SM, and (2) distinguish acute IS from SM and healthy controls. In all cases, combining different isodecoders in a single model yielded substantially higher diagnostic accuracies than individual isodecoders. By combining the tRFs that were identified in both the discovery and the validation cohort, we formulated a model consisting of three tRFs that had good diagnostic potential for differentiating IS from healthy controls, SM and ICH. As there were no ICH patients in the validation cohort, it was not surprising that the common tRF model showed only poor diagnostic accuracy for the differentiation between ICH and SM. In fact, this confirmed the validity of the data obtained from the discovery cohort of the MIRAS study in distinguishing between IS and ICH.

To our knowledge, this is the first study on sncRNAs that includes all acute stroke codes, thereby reflecting clinical practice. Furthermore, we demonstrate the diagnostic potential of tRFs in stroke patients, which, to our knowledge, has only been reported in in vitro and in vivo models before [9,10,11].

For stroke biomarkers to be of clinical use, rapid detection is crucial. A recent paper demonstrated that rapid detection of low concentrations of tRFs in plasma is indeed feasible [12]. We found that tRFs were also detectable in plasma depleted of extracellular vesicles, suggesting that circulating tRFs are mainly present outside vesicles. Therefore, we hypothesize that they can also be directly detected in whole blood, without needing to disrupt cellular or vesicular membranes. Importantly, these characteristics will absolve the need for labor- and time-intensive sample processing steps such as lysis, ultracentrifugation, RNA isolation and PCR, which is essential for point-of-care test development. Such a point-of-care test could potentially be used in the pre-hospital setting, for example in the ambulance or at the general practitioner. Thereby, the time to treatment could be drastically reduced, especially for ischemic strokes, which require reperfusion treatment as quickly as possible. Future studies need to confirm the feasibility, as well as the added diagnostic value of tRFs on top of clinical assessment (i.e., based on medical history, symptoms and neuroimaging), although the latter is known to be unreliable [13]. 

The prognostic potential of tRNA-derivatives in general has been shown by Ishida et al. in plasma collected from IS patients after seven days and has also been suggested in other diseases [4,14]. Apart from their potential as diagnostic biomarkers, it will therefore also be highly interesting to determine whether specific circulating tRFs have prognostic value for clinical outcome. Moreover, elucidating how tRFs are released into the circulation will be crucial to understand the pathophysiological link with stroke subtypes. Interestingly, a recent paper showed that tRNA-derived small RNAs in the brain may have a functional effect after experimental ICH induction in rats [15]. 

Our study is limited by the explorative design and small sample size. These limitations should be taken into account when interpreting the data. Due to the small sample size, we did not adjust for the fact that the groups were not completely matched for the vascular risk factors DM-II and hypertension, which may have confounded the results. Our results will therefore be validated in a larger cohort from the ongoing MIRAS study, allowing for multivariate analysis to adjust for vascular risk factors. Furthermore, the addition of tRFs as biomarkers to a clinical score could be investigated, for example using diagnostic measures such as the Net Reclassification Index or using likelihood-based methods [16,17]. Finally, our results have not yet been validated using an independent detection method such as RT-qPCR. Nonetheless, the IS and SM models yielded comparable AUCs in the validation cohort. As expected, the ICH model had much lower predictive value in the validation cohort, as this cohort did not include ICH patients. These findings provide additional support for our results and underline the specificity of the models for each stroke subtype. 

In conclusion, we identified tRFs as a promising novel biomarker class to distinguish between acute IS, ICH and SM. New detection technologies may facilitate swift translation into a point-of-care test that can eventually be implemented in clinical practice to reduce treatment delays and improve patient outcomes.

## 4. Materials and Methods

### 4.1. Study Design

This is a preplanned preliminary analysis of the MicroRNA in Acute Stroke (MIRAS) study, an ongoing prospective observational cohort study. Enrollment started on November 2018. This study is registered at AsPredicted.org with unique identifier #44134. Reporting was done in accordance with the STROBE Statement (see Supplementary Material) [18]. The study was conducted in accordance with the with the Declaration of Helsinki, the principles of Good Clinical Practice, the Dutch Agreement on Medical Treatment Act (WGBO) and the European General Data Protection Regulation and was approved by the Medical-Ethical Committee Leiden-Den Haag-Delft (P18.030; NL63060.058.17, 18 May 2018). Written informed consent was obtained from all patients or their legal guardian. 

### 4.2. Patient Selection

Consecutive stroke code patients aged 18 years or older who presented primarily at the emergency department of the Leiden University Medical Center within 6 h of symptom onset were included during office hours. Stroke was categorized according the American Heart Association/American Stroke Association [19]. Stroke mimics (SM) were defined as stroke code patients for whom a stroke as the underlying pathology was excluded. Additional inclusion criteria for stroke patients were admittance to the neurology ward and a NIHSS score of ≥4. Patients were excluded if they had an active malignant disease or used heparin within 24 h prior to symptom onset. In case of doubt about eligibility, blood samples were processed according to protocol. For all patients, the study coordinators (TTMN and MLvdB) verified that patients complied with the in- and exclusion criteria using the electronic patient record. Samples and clinical data of non-eligible patients or patients that did not provide informed consent were destroyed.

For this discovery analysis, we selected 27 patients (*n* = 8–10 per group) from a total of 68 eligible patients who were included between November 2018 and August 2019. Criteria for selection included technical quality of the sample (i.e., sufficient amount and absence of hemolysis) and a confirmed final diagnosis of IS, ICH or SM. One SM sample was excluded post hoc from our discovery cohort because of an uncertain onset-to-door time, leading to a final sample size of 26 (9 IS, 8 ICH and 9 SM). According to the standard stroke protocol, all patients underwent clinical assessment at presentation at the emergency department followed by non-contrast CT with CT-angiography if indicated. Final diagnosis was established three months after discharge by an experienced stroke neurologist (NDK) based on all available clinical and neuro-imaging data and blinded to blood biomarker outcome. 

### 4.3. Clinical Data

Clinical data, neuro-imaging data and diagnosis at admission, discharge and after three months were retrieved from electronic patient records. Clinical data included demographic characteristics, medical history, medication use and stroke severity as assessed with the NIHSS score. In case the NIHSS score was not available, this was reconstructed from neurological examination at admission by NIHSS certified research members with a validated algorithm as described previously [20]. In-hospital performance metrics included onset-to-door time. In case of a wake-up stroke, we included patients if the time of last seen well was within 6 h.

### 4.4. Sample Collection

Non-fasting venous blood was collected at the earliest possibility upon admission to the emergency department, prior to the administration of therapy or medication, using a 18G infusion needle in two 9 mL VACUETTE 9NC Coagulation sodium citrate tubes (Greiner Bio-One, Alphen aan den Rijn, the Netherlands). To prevent platelet activation and cell lysis, agitation of the blood collection tubes was kept to a minimum, tubes were kept vertical at all times after inverting carefully to mix the blood with the citrate buffer and they were delivered to the laboratory by one of the researchers, rather than through standard pneumatic tube delivery [21]. Blood was processed at room temperature within 60 min. Sodium citrate tubes were centrifuged for 15 min at 2500× *g*, after which plasma was transferred to a clean plastic 15 mL conical bottom centrifuge tube (Greiner) and centrifuged once more for 15 min at 2500× *g*. After each centrifugation step, plasma was pipetted off carefully from the top and at least 200 µL of plasma was left untouched in order to isolate platelet-poor plasma. Plasma aliquots were snap frozen in liquid nitrogen, then stored at −80 °C. Hemolysis was monitored visually and by spectrophotometry [22], as well as by RT-qPCR of the erythrocyte-specific microRNA miR-451a relative to the stable miR-23a-3p, prior to sequencing [23].

### 4.5. Ultracentrifugation and RNA-Sequencing

To deplete plasma of extracellular vesicles (EVs), 2.5 mL of plasma was thawed and diluted once with phosphate-buffer saline (PBS). The plasma was then depleted of EVs by two rounds of ultracentrifugation using a swinging-bucket rotor in a Beckman Coulter XE-90 instrument (Beckman Coulter, Woerden, The Netherlands). To remove large vesicles, samples were first centrifuged for 70 min at 4 °C at 17,500× *g*, using maximum acceleration (0) and slow deceleration (5). The upper 4.5 mL was transferred to a new tube and filled up to 5 mL with PBS, so that the final dilution of the plasma was approximately 2.2-fold. In the second step, samples were centrifuged for 70 min at 4 °C at 166,400× *g* to remove small vesicles, using the same acceleration and deceleration settings. Three fractions were separated: The top 0.5 mL, the middle 3.5 mL and the bottom 1 mL These fractions, as well as the bottom 0.5 mL that remained after the first round of ultracentrifugation, were aliquoted and snap-frozen on dry ice, then stored at –80 °C.

The upper 0.5 mL of EV-depleted plasma was sent to QIAGEN (Venlo, The Netherlands) for small RNA-sequencing (sRNA-seq). Briefly, RNA was isolated using the miRNeasy Serum/Plasma Kit (QIAGEN) according to the manufacturer’s instructions. Five microliters of total RNA per sample were used for library preparation using the QIAseq miRNA Library Kit (QIAGEN). Adapters containing unique molecular identifiers (UMIs) were ligated to the RNA, followed by reverse transcription. cDNA was amplified by 22 cycles of PCR and indices were added during the PCR. PCR samples were purified, and libraries were pooled in equimolar ratios. These pools were quantified using qPCR and sequenced on a NextSeq500 instrument (Illumina, San Diego, CA, USA) according to the manufacturer’s instructions. Raw data were de-multiplexed and FASTQ files were generated using the bcl2fastq software. Data quality was assessed using the FastQC tool [24].

Publicly available sRNA-seq data of an independent study cohort were retrieved from the NCBI Sequence Read Archive (project identifier SRP133275) [8]. In this cohort, the authors included 20 IS patients (≤24 h of symptom onset; mean 3.9 h ± 3.6 h) and 20 matched healthy controls. Briefly, the authors collected nonfasting blood samples at the emergency department in EDTA tubes using a 18G needle and tourniquet, after which platelet-poor plasma was isolated by two rounds of centrifugation (10 min at 2000× *g* followed by 15 min at 2500× *g*). RNA was isolated from the plasma using the miRCURY RNA Isolation Kit-Biofluids (Exiqon, Aarhus, Denmark); sRNA-seq libraries were prepared using the TruSeq Small RNA sample prepkit v2 (Illumina) and sequenced on an Illumina HighScan-SQ sequencer.

### 4.6. Data Analysis

Clinical data were analyzed using SPSS (v24.0, IBM, New York, NY, USA). Continuous data were presented as mean ± standard deviation (SD) if normally distributed, and as median with interquartile range (IQR) if skewed. Categorical data were presented as number and percentage. Continuous data were compared using One-way ANOVA or Kruskal–Wallis tests, as appropriate. Descriptive categorical data were analyzed using Fisher’s exact test.

Both sRNA-seq datasets were analyzed using the virtual machine of the sRNAtoolbox [25]. Briefly, the sRNAbench module was used to (i) trim the Illumina adapter sequence from the raw reads in the fastq files, (ii) identify and deduplicate reads based on UMIs, and (iii) map the reads to the human genome using bowtie. Several libraries were used for annotation, including miRbase, gtRNAdb, RNAcentral, and NCBI ncRNA and cDNA libraries. Then, the sRNAde module was used to investigate differential expression of various classes of small RNAs and variants. Data were visualized using Prism 8 (GraphPad) and R version 3.5.1.5 [26].

After mapping the sequencing reads, we calculated the levels of tRFs for each isodecoder by taking the sum of the multi-mapping adjusted total RPM (Reads Per Million, normalized to the total number of genome mapped reads). Isodecoder levels were then compared between the three groups by one-way ANOVA, followed by Tukey’s post hoc tests for pairwise comparisons. Least absolute shrinkage and selection operator (LASSO) regression analysis was performed to select optimal statistical models for the prediction of pathology (IS, ICH or SM), using the glmnet package in R [27]. Only isodecoders with a minimum expression of 1 read count in each sample and at least 10 RPM in at least 8 samples in our own dataset (resulting in 46 predictors) or at least 5 RPM in at least 20 samples in the independent dataset (resulting in 24 predictors) were considered in the LASSO analysis. Due to the small sample size per group in our own dataset, each group was compared to the other two groups using binomial analysis. The optimal model was defined as the model with the log (lambda) value that corresponded to the lowest binomial deviance. Generalized linear models were calculated for the optimal combination of predictors using the R stats package. Finally, using the pROC package in R, receiver operating characteric (ROC) curves were generated to assess diagnostic accuracy for each separate predictor, as well as for the generalized linear models of the combinations of predictors [28].

## Figures and Tables

**Table 1:** Patient baseline characteristics of the discovery cohort.

	Total No. Patients (*n* = 26)	Ischemic Stroke (*n* = 9)	Intracerebral Hemorrhage * (*n* = 8)	Stroke Mimics ^†^ (*n* = 9)	*p* Value
Age, mean (SD), *y*	71 (14)	76 (8)	71 (14)	65 (16)	0.25
Male sex, *n* (%)	15 (58)	6 (67)	3 (38)	6 (67)	0.43
**Medical history, *n* (%)**					
Ischemic stroke/TIA	8 (31)	3 (33)	3 (38)	2 (22)	0.87
Intracerebral hemorrhage	2 (8)	0 (0)	2 (25)	0 (0)	0.09
Atrial fibrillation	5 (19)	1 (11)	2 (25)	2 (22)	0.84
Diabetes Mellitus	5 (19)	1 (11)	4 (50)	0 (0)	0.02
Hyperlipidemia	12 (46)	6 (67)	3 (38)	3 (33)	0.38
Hypertension	15 (58)	6 (67)	7 (89)	2 (22)	0.02
**Medication use, *n* (%)**					
Oral anticoagulation	5 (19)	1 (11)	3 (38)	1 (11)	0.38
Antiplatelets	8 (31)	3 (33)	2 (25)	3 (33)	1.00
**Hospital admission**					
ODT, median (IQR), min	78 (57–116)	72 (45–144)	95 (63–203)	68 (60–102)	0.76
NIHSS ^‡^, median (IQR)	8 (4–18)	5 (4–19)	11 (6–18)	-	
LAVO ^§^, *n* (%)		4 (44)	-	-	-
**Neuro-imaging ^||^**					
Lesion confirmed, *n* (%)		8 (89)	8 (100)	-	
**Reperfusion therapy**					
IVT, *n* (%)	-	7 (78	-	-	-
EVT, *n* (%)	-	4 (44)	-	-	-
DNT, median (IQR), min	-	20 (17–40)	-	-	-
DGT, median (IQR), min	-	56 (51–65)	-	-	-

TIA: Transient ischemic attack; ODT: Onset-to-door time; NIHSS: National Institute of Health Stroke Scale; LAVO: Large anterior vessel occlusion; IVT: Intravenous thrombolysis; EVT: Endovascular treatment; DNT: Door-to-needle time in IVT-treated patients; DGT: Door-to-groin puncture time in EVT-treated patients. * Location of hemorrhage: 4 (50%) basal ganglia, 4 (50%) lobar. † Stroke mimics: 2 (22%) epilepsy, 5 (56%) acute vestibular syndrome (all with positive head impulse test), 1 (11%) intoxication, 1 (11%) partial oculomotor nerve palsy. ‡ Stroke only (i.e., ischemic stroke and intracerebral hemorrhage). § Occlusion of the intracranial carotid artery(-tandem) (ICA/ICA-T), middle cerebral artery (M1/M2), or anterior cerebral artery (A1/A2). || Computed tomography, -angiography/-perfusion, magnetic resonance imaging.

## Data Availability

The sequencing data presented in this study are openly available in the European Nucleotide Archive (ENA) at EMBL-EBI under accession number PRJEB40899. The clinical data presented in this study are available on request from the corresponding author. These data are not publicly available due to privacy regulations.

## Supplementary Materials

The following are available online at https://www.mdpi.com/1422-0067/22/1/135/s1, STROBE checklist. Table S1: Sum of tRF levels for each isodecoder. Table S2: Diagnostic parameters determined by ROC analysis on generalized linear models of combinations of isodecoders.
