# Supplementary material for: Circulating tRNA Fragments as a Novel Biomarker Class to Distinguish Acute Stroke Subtypes
Source: Int J Mol Sci. 2020 Dec 24;22(1):135. doi: 10.3390/ijms22010135 (PMC7796003; doi:10.3390/ijms22010135)
Supplement: Supplementary file 1 [file ijms-22-00135-s001.pdf]

International Journal of
Molecular Sciences

MDPI

SUPPLEMENTARY MATERIAL

Circulating tRNA fragments as a novel biomarker class to distinguish acute stroke subtypes

T Truc My Nguyen, MD; M Leontien van der Bent, PhD; Marieke JH Wermer, MD, PhD; Ido R van den Wijngaard, MD, PhD; Erik W van Zwet, PhD; Bas de Groot, MD, PhD; Paul HA Quax, PhD; Nyika D Kruyt, MD, PhD; A Yaël Nossent, PhD.

STROBE Statement—Checklist of items that should be included in reports of ***cohort studies***

	Item No	Recommendation	Page No
**Title and abstract**	1	(*a*) Indicate the study's design with a commonly used term in the title or the abstract	1
		(*b*) Provide in the abstract an informative and balanced summary of what was done and what was found	1
**Introduction**			
Background/rationale	2	Explain the scientific background and rationale for the investigation being reported	2
Objectives	3	State specific objectives, including any prespecified hypotheses	2
**Methods**			
Study design	4	Present key elements of study design early in the paper	10
Setting	5	Describe the setting, locations, and relevant dates, including periods of recruitment, exposure, follow-up, and data collection	10-11
Participants	6	(*a*) Give the eligibility criteria, and the sources and methods of selection of participants. Describe methods of follow-up<br>(*b*) For matched studies, give matching criteria and number of exposed and unexposed	10
Variables	7	Clearly define all outcomes, exposures, predictors, potential confounders, and effect modifiers. Give diagnostic criteria, if applicable	11
Data sources/ measurement	8*	For each variable of interest, give sources of data and details of methods of assessment (measurement). Describe comparability of assessment methods if there is more than one group	11
Bias	9	Describe any efforts to address potential sources of bias	9
Study size	10	Explain how the study size was arrived at	10
Quantitative variables	11	Explain how quantitative variables were handled in the analyses. If applicable, describe which groupings were chosen and why	13-14
Statistical methods	12	(*a*) Describe all statistical methods, including those used to control for confounding<br>(*b*) Describe any methods used to examine subgroups and interactions<br>(*c*) Explain how missing data were addressed<br>(*d*) If applicable, explain how loss to follow-up was addressed<br>(*e*) Describe any sensitivity analyses	13-14
**Results**			
Participants	13*	(a) Report numbers of individuals at each stage of study—eg numbers potentially eligible, examined for eligibility, confirmed eligible, included in the study, completing follow-up, and analysed	8

		(b) Give reasons for non-participation at each stage<br>(c) Consider use of a flow diagram	
Descriptive data	14*	(a) Give characteristics of study participants (eg demographic, clinical, social) and information on exposures and potential confounders<br>(b) Indicate number of participants with missing data for each variable of interest<br>(c) Summarise follow-up time (eg, average and total amount)	3
Outcome data	15*	Report numbers of outcome events or summary measures over time	3s
Main results	16	(*a*) Give unadjusted estimates and, if applicable, confounder-adjusted estimates and their precision (eg, 95% confidence interval). Make clear which confounders were adjusted for and why they were included<br>(*b*) Report category boundaries when continuous variables were categorized<br>(*c*) If relevant, consider translating estimates of relative risk into absolute risk for a meaningful time period	3-7
Other analyses	17	Report other analyses done—eg analyses of subgroups and interactions, and sensitivity analyses	N.A.
**Discussion**			
Key results	18	Summarise key results with reference to study objectives	8
Limitations	19	Discuss limitations of the study, taking into account sources of potential bias or imprecision. Discuss both direction and magnitude of any potential bias	9
Interpretation	20	Give a cautious overall interpretation of results considering objectives, limitations, multiplicity of analyses, results from similar studies, and other relevant evidence	8-9
Generalisability	21	Discuss the generalisability (external validity) of the study results	8
**Other information**			
Funding	22	Give the source of funding and the role of the funders for the present study and, if applicable, for the original study on which the present article is based	15

*Give information separately for exposed and unexposed groups. An Explanation and Elaboration article discusses each checklist item and gives methodological background and published examples of transparent reporting. The STROBE checklist is best used in conjunction with this article (freely available on the Web sites of PLoS Medicine at http://www.plosmedicine.org/, Annals of Internal Medicine at http://www.annals.org/, and Epidemiology at http://www.epidem.com/). Information on the STROBE Initiative is available at http://www.strobe-statement.org.

Supplementary Tables

Table S1. Sum of tRF levels for each isodecoder. Mean and standard deviation of the sum of the multi-mapping adjusted total RPM. One-way ANOVA was used to test for differences between group means.

	ICH (N=8)		IS (N=9)		SM (N=9)		
**Isodecoder**	**Mean**	**SD**	**Mean**	**SD**	**Mean**	**SD**	**p value**
SerGCT	112.2	44.0	77.4	18.7	67.1	23.0	0.0138
LeuCAG	28.8	17.6	47.5	21.3	53.5	15.6	0.0301
AsnGTT	132.0	98.5	62.2	15.3	64.0	42.3	0.0438
ArgTCG	45.2	33.1	24.4	11.3	21.0	7.6	0.0440
ValCAC	145.0	56.7	128.9	30.2	179.0	47.3	0.0800
ProAGG	77.6	83.3	30.1	9.8	32.9	18.7	0.0959
IleAAT	204.3	106.5	140.3	35.8	145.7	48.0	0.1302
LeuTAA	48.4	20.0	32.9	11.1	37.7	15.3	0.1385
ThrTGT	47.3	26.7	33.0	14.5	30.1	13.3	0.1567
GlyCCC	698.2	516.9	392.1	109.8	701.9	450.1	0.1894
HisGTG	49.4	21.3	53.5	27.3	68.7	22.1	0.2270
GlyTCC	220.4	434.8	41.2	19.0	44.3	24.1	0.2420
SerACT	7.1	4.9	9.9	8.8	13.6	8.9	0.2544
ProCGG	37.1	41.3	18.0	5.4	21.3	14.3	0.2551
GlyGCC	1016.6	744.5	535.6	239.0	1262.2	1376.3	0.2560
ArgTCT	162.1	23.9	134.9	46.3	136.6	52.0	0.3693
ValAAC	77.2	48.5	51.8	18.8	69.6	43.0	0.3877
TyrATA	6.4	3.2	8.4	3.5	6.7	2.8	0.3945
ArgCCG	21.3	12.9	15.4	6.4	18.1	7.3	0.4208
AlaAGC	156.7	55.4	136.2	22.5	136.3	26.1	0.4361
CysGCA	454.3	475.0	271.7	120.0	352.8	196.5	0.4566
AspGTC	932.5	325.4	783.0	108.1	888.2	286.6	0.4681
LeuAAG	192.8	165.6	231.8	122.2	272.2	101.9	0.4706
ThrCGT	27.2	14.4	20.8	6.4	24.7	10.5	0.4723
AlaTGC	152.5	68.1	168.4	39.8	185.9	60.0	0.4894
Undet???	6.2	3.5	8.2	3.7	7.7	3.8	0.5230
LeuTAG	74.2	48.2	79.1	42.0	95.8	36.9	0.5441
ProTGG	259.5	281.2	178.8	78.6	184.2	59.3	0.5487
TyrGTA	90.8	23.1	84.8	21.5	97.3	26.9	0.5525
LysCTT	464.4	436.5	343.7	158.2	342.3	57.2	0.5563
LysTTT	337.0	162.9	270.8	84.8	308.4	124.0	0.5620
SerCGA	61.4	51.6	65.7	47.7	45.6	17.3	0.5639
GluTTC	185.4	201.8	172.6	79.5	236.3	103.5	0.5829
SupTTA	18.5	10.4	14.1	3.4	17.0	11.0	0.5928
MetCAT	117.4	66.7	99.8	27.3	98.9	15.4	0.5930

TrpCCA	8.7	6.2	12.1	10.5	9.2	4.1	0.6025
GlnTTG	21.0	38.2	10.2	6.8	15.2	9.6	0.6130
SerAGA	96.2	37.5	102.4	21.7	90.7	26.3	0.6956
AlaCGC	51.8	44.8	41.8	8.1	50.4	18.1	0.7138
ValTAC	304.8	151.4	263.2	83.0	280.2	94.4	0.7465
GluCTC	278.0	110.0	278.1	115.2	249.1	44.7	0.7627
LeuCAA	59.1	36.1	50.6	14.6	55.8	19.6	0.7705
GlnCTG	11.6	8.5	9.7	5.8	11.3	4.6	0.8017
PheGAA	110.6	38.2	102.4	32.8	101.7	38.3	0.8605
ArgACG	20.7	27.2	16.5	10.1	16.8	10.6	0.8634
SerTGA	72.0	12.0	75.4	16.1	72.8	21.2	0.9083
SeC(e)TCA	31.9	23.1	31.1	23.6	34.1	21.3	0.9586
ArgCCT	28.5	10.6	29.1	7.9	28.0	8.0	0.9662

Table S2. Diagnostic parameters determined by ROC analysis on generalized linear models of combinations of isodecoders.

	Sensitivity	Specificity	AUC (95% CI)
**Optimal LASSO models (discovery cohort; Figure 1C)**			
ICH vs other groups (ICH model)[*]	1.000	0.889	0.986 (0.953-1.000)
IS vs other groups (IS model)[†]	0.889	0.941	0.967 (0.907-1.000)
SM vs other groups (SM model)[‡]	0.765	1.000	0.928 (0.832-1.000)
**Previous models (validation cohort; Figure 2)**			
ICH model[*]	0.550	0.950	0.728 (0.561-0.894)
IS model[†]	0.800	0.900	0.870 (0.756-0.984)
SM model[‡]	0.800	0.850	0.885 (0.781-0.989)
**New models (validation cohort; Figure 3A and 3B)**			
Optimal LASSO model[§]	1.000	1.000	1.000 (1.000-1.000)
Common tRF model[‖]	0.800	0.900	0.875 (0.759-0.991)
**Common tRF model[‖] (discovery cohort; Figure 3C)**			
ICH vs SM[‖]	0.750	0.667	0.653 (0.359-0.947)
ICH vs IS[‖]	0.625	1.000	0.847 (0.656-1.000)
IS vs SM[‖]	1.000	1.000	1.000 (1.000-1.000)

*LeuCAG, ArgTCG, LeuTAA and SerGCT; †ValCAC, ThrCGT, LeuCAG and GlyCCC; ‡TyrGTA, ValCAC, LeuCAG, SerGCT and SerACT; §TyrGTA, ValCAC, MetCAT, ThrCGT, HisGTG, AlaTGC, LysCTT, TyrATA and AlaAGC; ||ValCAC, TyrGTA and ThrCGT.
